# The role of oxidative stress in degeneration of the neuromuscular junction in amyotrophic lateral sclerosis

**DOI:** 10.3389/fncel.2014.00131

**Published:** 2014-05-13

**Authors:** Eveliina Pollari, Gundars Goldsteins, Geneviève Bart, Jari Koistinaho, Rashid Giniatullin

**Affiliations:** ^1^Molecular Brain Research Laboratory, Department of Neurobiology, A. I. Virtanen Institute for Molecular Sciences, University of Eastern FinlandKuopio, Finland; ^2^Experimental Neurology - Laboratory of Neurobiology, Department of Neurosciences, Vesalius Research Center, KULeuven – University of LeuvenLeuven, Belgium; ^3^Cell Biology Laboratory, Department of Neurobiology, A. I. Virtanen Institute for Molecular Sciences, University of Eastern FinlandKuopio, Finland; ^4^Laboratory of Neurobiology, Department of Physiology, Kazan Federal UniversityKazan, Russia

**Keywords:** ALS, neuromuscular junction, ROS, oxidative stress, neurodegeneration

## Abstract

Amyotrophic lateral sclerosis (ALS) is characterized by the progressive loss of motoneurons and degradation of the neuromuscular junctions (NMJ). Consistent with the dying-back hypothesis of motoneuron degeneration the decline in synaptic function initiates from the presynaptic terminals in ALS. Oxidative stress is a major contributory factor to ALS pathology and affects the presynaptic transmitter releasing machinery. Indeed, in ALS mouse models nerve terminals are sensitive to reactive oxygen species (ROS) suggesting that oxidative stress, along with compromised mitochondria and increased intracellular Ca^2+^ amplifies the presynaptic decline in NMJ. This initial dysfunction is followed by a neurodegeneration induced by inflammatory agents and loss of trophic support. To develop effective therapeutic approaches against ALS, it is important to identify the mechanisms underlying the initial pathological events. Given the role of oxidative stress in ALS, targeted antioxidant treatments could be a promising therapeutic approach. However, the complex nature of ALS and failure of monotherapies suggest that an antioxidant therapy should be accompanied by anti-inflammatory interventions to enhance the restoration of the redox balance.

## NMJ as a vulnerable target of ALS (Dying Back hypothesis)

**Figure 1:**
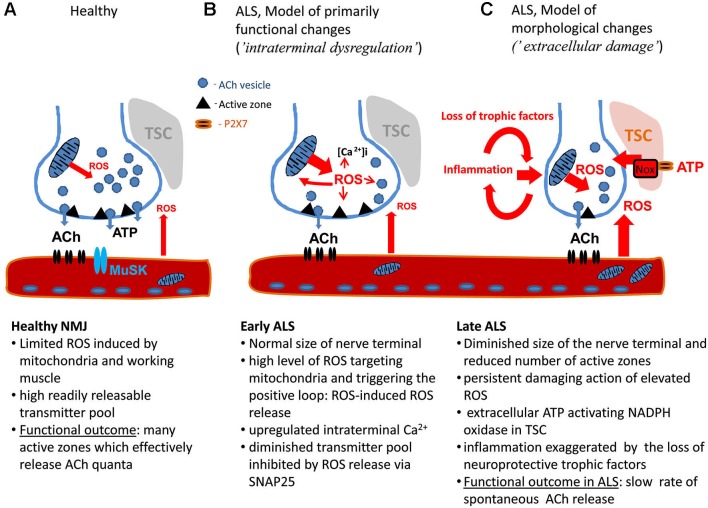
**The model of motor nerve terminal dysregulation in ALS**. **(A)** Healthy NMJ. **(B)** Pathological changes in NMJ during early stage of ALS. **(C)** Pathological changes in NMJ during late stage of ALS. ACh: Acetyl choline, MuSK: muscle-specific kinase, NMJ: Neuromuscular junction, ROS: Reactive oxygen species, TSC: Terminal Schwann Cells.

Temporal analysis of axon and neuromuscular junction (NMJ) degeneration in sporadic ALS (sALS) and mouse mutant SOD1 (mSOD1) cases indicate that motoneuron pathology begins distally from the synaptic area (Figure 1) markedly earlier than clinical symptoms and proceeds towards soma in a retrograde dying back manner (Fischer et al., 2004; Rocha et al., 2013). Impaired axonal transport, Ca^2+^ imbalance and mitochondria dysfunction drive the axonal degeneration, and eventually lead to dying of the neuron (Fischer-Hayes et al., 2013).

Figure 1 shows the principal structure of the NMJ including the presynaptic machinery restricted to active zones (AZ) releasing acetyl choline (ACh) in quantal manner and postsynaptic structures consisting of densely packed ACh receptors linked to the muscle-specific kinase (MuSK), agrin and other molecules involved in NMJ maturation and maintenance (reviewed in Shi et al., 2012). Thus, the dysfunction of the neuromuscular transmission can originate from the presynaptic site or from disorganized postsynaptic density. Notably, the motor nerve terminals are covered by the Terminal Schwann Cells (TSC) which can contribute to ALS progression.

In mSOD1 mice many motor terminals of the diaphragm muscle show a number of dysfunctional changes in the early disease stage (Naumenko et al., 2011). Muscle fibers are proposed to initiate the early changes leading to ALS progression (Wong and Martin, 2010). However, our results indicate that in the NMJ of ALS mice the presynaptic machinery is affected first (Naumenko et al., 2011). There is a noticeable variation in the probability of transmitter release between synapses, suggesting different degeneration rates of synapses. At the early symptomatic phase, only a few synapses have compromised function. Presumably, early in the disease course, the proportion of damaged synapses is low allowing compensation of the lost function by the healthy ones. Interestingly, during the pre-symptomatic stage enhanced neuromuscular transmission can be observed before the occurrence of the marked decline during the symptomatic phase, possibly due to compensatory mechanisms against the initial degeneration (Rocha et al., 2013).

Indeed, while some axon branches degenerate in ALS, others show sprouting thus compensating for lost synapses (Schaefer et al., 2005). Supporting the regenerating axons provides a therapeutic opportunity for maintaining innervation. However, as the disease progresses the proportion of damaged synapses increases and the sparse functional synapses cannot mediate synaptic transmission anymore. In mouse models of ALS axons of fast-fatiguable motoneurons are affected synchronously in hindlimbs, long before symptoms appear, whereas axons of slow motoneurons are more resistant. Thus it is possible that ALS involves predictable, selective vulnerability patterns of NMJs by physiological subtypes of axons, where NMJs of the resistant axons partially assume compensatory functions (Pun et al., 2006; Dibaj et al., 2011).

In some mSOD1 mouse models, oxidative stress appears to originate from distal muscles before the disease onset (Kraft et al., 2007). Reactive oxygen species (ROS) affect synaptic transmission by inhibiting transmitter release. Increasing ROS levels further inhibit NMJ function in spite of already elevated level of oxidative stress (Naumenko et al., 2011). This suggests that oxidative damage could start in peripheral tissues and proceed retrogradely to neurons. Skeletal muscle targeted expression of mSOD1 provokes motor deficits, but at a rather late age and without evident effect on the life expectancy (Wong and Martin, 2010). In this particular model, the muscle pathology is accompanied by NMJ abnormalities and distal motoneuron axonopathy. Initiation of the motoneuron degeneration by muscle cells supports the hypothesis of dying-back pathogenesis where the neurodegeneration starts from deficits in muscle and NMJs and proceeds from distal axons towards soma leading to apoptosis of motoneurons (Fischer et al., 2004; Dupuis and Loeffler, 2009).

## Presynaptic part of the NMJ as the main sensitive part reacting to oxidative stress

Measurements from the diaphragm muscle of G93A-SOD1 mice have revealed a dramatic reduction in the frequency of miniature end-plate potentials (MEPPs) during the early symptomatic stage (Naumenko et al., 2011). Remarkably, no changes in the amplitude of MEPPs were observed, indicating purely presynaptic decline in the synaptic transmission. The amplitude of single evoked EPPs remained unchanged suggesting vulnerability of spontaneous quantal transmitter release from nerve terminals.

This phenotype (selective depression of MEPPs with little affected EPPs) largely resembles the inhibitory action of ROS on transmitter release at the frog NMJ: exogenous H_2_O_2_ elicits a strong inhibition of spontaneous release with limited effect on EPPs (Giniatullin and Giniatullin, 2003). NMJ impairment in ALS could therefore be produced by mechanisms similar to those, which affect synapses damaged by oxidative stress. Recent studies revealed distinct mechanisms underlying spontaneous versus evoked transmitter release (Maximov et al., 2007; Pang et al., 2011; Melom et al., 2013). Soluble N-ethylmaleimide-sensitive factor attachment protein receptors (SNARE) protein, Snap25, was identified as the main targets of ROS at the presynaptic level (Giniatullin et al., 2006) and could be one of the targets of ROS inhibiting transmitter release (Figure 1B).

Apart from ROS, another interesting candidate contributing to the damage of NMJ in ALS is extracellular ATP. ATP, the major co-transmitter of ACh at the NMJ (Redman and Silinsky, 1994), can produce a strong inhibitory action on transmitter release (Giniatullin and Sokolova, 1998) via ROS induction (Giniatullin et al., 2005; Sciancalepore et al., 2012). This mechanism could contribute to the motor nerve terminal dysregulation in ALS (Figure 1B) or, when applied persistently, to ATP-driven neurodegeneration of NMJ (Figure 1C). This view is consistent with recent data showing that extracellular ATP, operating via cytotoxic P2X7 receptors could largely regulate immune function and inflammatory responses (Volonté et al., 2012). Notably, TSC also express P2X7 receptor (Grafe et al., 1999; Colomar et al., 2003; Nobbio et al., 2009). Whereas accumulating evidence suggest that Schwann cells can contribute to ALS (De Winter et al., 2006; Gorlewicz et al., 2009; Lobsiger et al., 2009; Chen et al., 2010), the role of myelinating versus non-myelinating TSCs in ALS however requires, further studies (Turner et al., 2010).

We propose that the early damage to the NMJ in ALS is due to intraterminal dysregulation of nerve terminals without essential changes in their morphology (Figure 1B). Underlying mechanisms probably include dysfunctional mitochondria, intracellular Ca^2+^ and ROS. Elevated intraterminal Ca^2+^ can eventually support enhanced Ca^2+^-dependent evoked release during early stage of ALS (Rocha et al., 2013). The other model (Figure 1C), applicable to the later stage of ALS, suggests that the main damage results from the accumulation of toxic ROS, inflammatory factors, including glial transmitters from local Schwann cells and invasive immune cells, and absence of neuroprotective trophic factors. However, these two scenarios most likely co-exist within the same muscle during ALS progression providing a heterogeneous picture of morphological and functional changes (Rocha et al., 2013) and resulting in the pitfalls of the monotherapy in this disease.

## Axonal transport, presynaptic mitochondria and ROS-induced ROS release

Correct spatial distribution of mitochondria within a cell is an instrumental prerequisite for normal physiology. In neurons, mitochondria are subjected to both anterograde and retrograde axonal transport, which in case of motoneurons covers substantial distances. The transport of mitochondria in axons is driven along microtubules by kinesin and dynein motors (Pilling et al., 2006).

Accumulation of mitochondria at presynaptic nerve terminals of motoneurons is thought to support synaptic function through ATP production and partially take part in Ca^2+^ buffering during neurotransmission (Figure 1; Chouhan et al., 2010). Mitochondria are connected to the presynaptic membrane by a complex cytoskeletal superstructure, which is connected with nerve terminal filamentous linkages between synaptic vesicles, providing polarized organization for mitochondrial crista structures (Perkins et al., 2010). Defects in neuronal mitochondrial morphology and axonal transport have been demonstrated in primary neuronal cultures from ALS model animals (De Vos et al., 2007; Magrané et al., 2012). Importantly, these abnormalities are also observed in vivo in both SOD1 and TDP43 ALS mouse models, indicating that they are common denominators of different genetic forms of ALS (Magrané et al., 2014).

The high order of mitochondrial organization at presynaptic nerve terminals implies their participation in coordinated responses to various stimuli. One of the fundamental oxidative stress responses in mitochondria is mitochondrial permeability transition (MPT) pore opening, followed by sudden collapse of membrane potential and burst of ROS production, which might contribute to the spreading of MPT in bystanding mitochondria, and lead to the effect known as ROS-induced ROS release (Zorov et al., 2000). The latter can contribute to the functional dysregulation within the nerve terminal during the early stage of ALS (Figure 1B). Our studies have demonstrated that SOD1 activity is increased in ALS animal spinal cord mitochondria, and causes elevated hydroperoxide production (Ahtoniemi et al., 2008; Goldsteins et al., 2008). Augmented hydroperoxide flux from presynaptic mitochondria might contribute not only to reduced probability of quatal ACh release but also to the desynchronization of neurotransmitter release at NMJ (Tsentsevitsky et al., 2013) which would additionally diminish synaptic efficacy (Figure 1C). Apart from presynaptic location, muscle mitochondria and activity of NADPH oxidase in TSCs can serve as an additional source of ROS (Figure 1).

## Neuroinflammation, immune cells and oxidative stress in spinal cord in ALS

Oxidative stress, such as free radical damage and abnormal free radical metabolism, is evident in sALS and fALS patients (Shaw et al., 1995; Ferrante et al., 1997; Smith et al., 1998; Chang et al., 2008). The aberrant activity of mSOD1 leads to oxidative damage (Wiedau-Pazos et al., 1996; Crow et al., 1997) and other ALS-linked proteins, such as mutant TDP-43, promote oxidative stress in a motoneuron cell line (Duan et al., 2010). Excitotoxicity and oxidative stress caused by astrocytes arises from aberrant glutamate receptor function which leads to misregulated glutamate homeostasis (Rothstein et al., 1992). Oxidative stress promotes tissue damage by exacerbating and interacting with other pathological events that promote motoneuron degeneration.

Inflammation, which is an additional source of ROS, is evident in ALS patients and mSOD1 mice; microglia are activated and proliferating whereas the T cells and dendritic cells infiltrate into the spinal cord (Engelhardt et al., 1993; Henkel et al., 2004, 2006). Moreover, there is marked increase in pro-inflammatory cytokines and enzymes, such as interleukin-6 (IL-6), monocyte chemoattractant protein-1 (MCP-1), IL-8, and cyclooxygenase-2 (Cox-2) (Sekizawa et al., 1998; Almer et al., 2001, 2002; Elliott, 2001; Hensley et al., 2002; Kuhle et al., 2009). Astrocytes expressing mSOD1 are also prone to exhibit an activated pro-inflammatory state (Hensley et al., 2006; Di Giorgio et al., 2008; Marchetto et al., 2008). Activated pro-inflammatory M1 microglia cause ROS and glutamate excitotoxicity induced motoneuron injury and death (Zhao et al., 2004). MSOD1 induced oligodendrocyte dysfunction drives demyelination in the spinal cord and accelerates motoneuron degeneration (Kang et al., 2013).

Immune responses are also activated in peripheral tissues of ALS patients (Mantovani et al., 2009). Regulatory T (Treg) cells lower neuroinflammation through microglia by inducing secretion of anti-inflammatory cytokines IL-10 and transforming growth factor-β (TGF-β; Kipnis et al., 2004; Mantovani et al., 2009). In ALS patients, elevated levels of Treg cells and CD4 T cells in blood correlate with slow disease progression (Beers et al., 2011). T cell infiltration in the spinal cord in mSOD1 mice is amplified during the presymptomatic stage and the number of T cells in the spinal cord increases as the disease progresses (Beers et al., 2008; Chiu et al., 2008). The spinal cord T cell population is mainly composed of helper CD4 cells. The proportion of cytotoxic CD8 becomes prominent at the end-stage. This supports the assumption that during the early stages of the disease, there is a systemic combat to maintain neuroprotective responses, but as the disease aggravates, the immune response shifts towards cytotoxic.

Macrophages infiltrate ventral spinal roots, peripheral motor nerves and skeletal muscles in ALS mouse models (Chiu et al., 2009; Graber et al., 2010). The role of macrophages in affected tissues in ALS mice appears to be the phagocytic removal of debris from axonal degeneration. Thus, activated macrophages could contribute to ROS production in axons and muscle in ALS and along with other inflammatory agents participate in triggering of sprouting in nerve terminals. However, in ALS mice the majority of activated macrophages accumulated within fascicles of motoneurons in the peripheral tissues and were only rarely found adjacent to end-plate of NMJs. It is therefore unlikely, that macrophages directly contribute to oxidative damage of NMJs in ALS.

Interestingly, in ALS motoneurons in the brainstem oculomotor nuclei and Onuf’s nucleus in the sacral spinal cord are preserved and selective vulnerability seems to be related to oxidative stress. Reduced capability to maintain calcium homeostasis and disturbed mitochondrial function predispose specific motoneurons to degeneration in ALS (Vanselow and Keller, 2000; Jaiswal and Keller, 2009). In addition, the most vulnerable motoneurons are more prone to endoplasmic reticulum stress and exhibit increased susceptibility to excitotoxicity (Saxena et al., 2009; Brockington et al., 2013).

## Gender dependence of ALS and oxidative stress

ALS affects men more than women, with earlier age of onset for men as well a tendency for spinal initiation of the disease whereas in women it is more commonly bulbar (McComb and Henderson, 2010). Most of these features were also observed in mSOD1 animals (Veldink et al., 2003; Suzuki et al., 2007). Gender specific differences are also detected at the synapses: Synaptic vesicle release being more frequent in females’ end-plate with impairment only observed in males (Naumenko et al., 2011). Specific interneurons control motoneuron excitability via specialized cholinergic synaptic boutons: C-boutons. In ALS there is no gender difference in the number of C-boutons, but their size is bigger in male mSOD1 mice (Herron and Miles, 2012).

The most obvious explanation for gender differences is a protective effect of estrogen. ROS damage in muscle is limited in young women. Even aging women have significantly less lipid peroxidation, protein carbonylation and mitochondrial DNA damage than men (Pansarasa et al., 2000). Several estrogen-controlled pathways might protect females against fast neuromuscular degeneration in ALS, for instance estrogen-mediated cyclophilin D prevention of mitochondrial calcium overload (Kim et al., 2012). However, experiments with ovariectomized mSOD1 mice or rats with and without supplemental 17β-estradiol do not support the idea that estrogen could explain the gender differences in ALS (Choi et al., 2008; Hayes-Punzo et al., 2012).

Additional evidence for gender differences in the ROS balance are coming to light, for instance, lower blood level of uric acid (UA) were observed in ALS patients (Keizman et al., 2009). UA, a scavenger of NO radical and superoxide, reduces damage to cells, by preventing protein nitration on tyrosine residues by peroxynitrites, and higher level of UA in blood increases likelihood of longer survival in men (Paganoni et al., 2012).

Gender differences are also striking in the response to treatments. Examples of therapeutic approaches with gender bias are specific inhibition of spinal cord microglial P2X7, which appears to increase the duration of life without affecting the age of symptom onset in male (Cervetto et al., 2013) and G-CSF treatment which delays disease progression in male mSOD1 mice (Pitzer et al., 2008; Naumenko et al., 2011; Pollari et al., 2011).

## Promising therapeutic approaches and challenges of the antioxidant therapy in ALS

Several molecules with antioxidant capabilities have failed in clinics after showing promise in animal models (Gordon, 2013; Musarò, 2013; Pandya et al., 2013; Sreedharan and Brown, 2013). Still, riluzole is the only approved drug that delays the progression of ALS.

The translational failures in ALS can be explained by the same arguments as in other neurodegenerative diseases: (a) animal models represent only a fraction of genetic variations among ALS patients and do not model well sALS; (b) the preclinical studies are characterized by inadequate randomization, blinding, statistical power, control cohorts and consideration of comorbidities; and (c) optimal dosing and administration route in clinics are unknown, flaws in patient stratification or identification of proper patients, and insufficient samples size (Ubogu, 2012; Planas, 2013).

Perhaps the most important reason for the translation block is that by the time of diagnosis, ALS has already progressed too far, making prevention of further deterioration challenging. The late diagnosis allows multiple disease mechanisms to accelerate their contributions to motoneuron death overriding the regenerative mechanisms and preconditions. Also, nerve terminal retraction, axonal degeneration and eventual neuronal death may take weeks or months before their deteriorating effects become clinically noticeable (Coleman and Freeman, 2010; Sreedharan and Brown, 2013). The late time of diagnosis is an especially important concern for protection of NMJ functions, as NMJ degeneration is among the earliest pathological alterations in ALS.

During the presymptomatic phase of the ALS, oxidative stress may be triggered by increased production of superoxide and nitric oxide in neurons, central and peripheral glia and even in muscle cells. Also, levels of a major intracellular antixodiant, glutathione, reduce early in ALS tissues. At the same time, blood-spinal cord barrier appears to become leaky and infiltration of inflammatory cells into the spinal cord, motor nerves and muscles contributes to oxidative stress present prior to the disease onset (Henkel et al., 2004; Chang et al., 2008; Chiu et al., 2009; Chen et al., 2010; Halter et al., 2010; Dibaj et al., 2011; Drechsel et al., 2012; Winkler et al., 2014). In fact, several findings favor the idea of linking ALS therapy to the oxidative stress-related degeneration of NMJ. First, normal SOD1 activity is required for maintenance of NMJ function in aged rodents (Sakellariou et al., 2014) and, in a zebrafish model expressing mSOD1 in physiological levels, NMJ has increased susceptibility to oxidative stress showing early morphological alterations (Da Costa et al., 2014). Second, even though pathological changes in synapses and axons occur early during the ALS pathogenesis, these self-destructive mechanisms could be delayed by correcting molecular environment (Sreedharan and Brown, 2013). Third, the NOX-mediated increase in superoxide production takes place in neural cells in mSOD1 models of ALS (Harraz et al., 2008; Jaronen et al., 2013). Even though is it not known whether NOX is expressed in Schwann cells around NMJs, NOX could well contribute to oxidative deterioration of NMJ in ALS, as muscle cells express various isoforms of NOX. While it is unclear whether ALS-linked mutations or conditions in sALS could trigger activation of NOX in skeletal muscles, inhibitors of NOX activation are known to provide protection in ALS models. Considering that NOX activation pathway is a readily druggable target, the role of NOX in NMJ degeneration in ALS models is worth exploring (Sullivan-Gunn and Lewandowski, 2013). Finally, it is of interest that Vitamin D, an essential dietary vitamin with multiple physiological functions, has been demonstrated to influence several aspect of ALS pathology, including skeletal muscle strength and oxidative stress (Gianforcaro and Hamadeh, 2014).

Overall, recent research on NMJ, oxidative stress and inflammation in ALS models warrant further preclinical investigation of the possibility of developing an ALS therapy by targeting the signaling pathways of NMJ dysfunction, provided that early diagnosis of ALS and biomarkers for NMJ dysfunctions become available (Figure 1). While keeping in mind the previous failures in clinical trials for ALS, it is evident that multiple mechanisms, including oxidative stress in the center, contribute to ALS pathogenesis. The concept of combination therapy is not novel in the field of neurodegenerative diseases, but it is still a valid approach once most of the key targets of the disease mechanisms, including oxidative stress become identified.

## Conflict of interest statement

The authors declare that the research was conducted in the absence of any commercial or financial relationships that could be construed as a potential conflict of interest.
